# Prenatal antibiotic exposure in pregnancy and early childhood socioemotional development

**DOI:** 10.1002/jcv2.12066

**Published:** 2022-03-29

**Authors:** Adi Fish‐Williamson, Jennifer Hahn‐Holbrook, Mark Hobbs, Jan Wallander, Susan M. B. Morton

**Affiliations:** ^1^ Department of psychological sciences University of California Merced Merced California USA; ^2^ Auckland City Hospital Auckland New Zealand; ^3^ Growing Up in New Zealand, Centre for Longitudinal Research – He Ara Ki Mua The University of Auckland Auckland New Zealand

**Keywords:** antibiotics, prenatal, socioemotional development

## Abstract

**Background:**

Antibiotic exposure in pregnancy is associated with reduced microbiome diversity in the infant gut. Given that recent research has shown that early microbiome health can impact child socioemotional development, we examined the relationship between prenatal antibiotic exposure in pregnancy and childhood socioemotional developmental outcomes using a large, nationally representative longitudinal dataset.

**Methods:**

A sample of 4800 diverse families were assessed from the population cohort of the Growing Up in New Zealand Study (GUiNZ), which prospectively follows children starting in the last trimester of pregnancy into early childhood. Socioemotional development was measured using a composite score derived from seven commonly used socioemotional tasks administered between 9 months and 4.5 years of child age, addressing emotional expression understanding, regulation of emotions and behavior, and social problem solving and relationship skills. A national comprehensive pharmaceutical database was used to determine children's prenatal antibiotic exposure. Multivariate linear regressions models were used to examine the effects of the timing (trimester) and dosage (number of courses) of prenatal antibiotic exposure on socioemotional development, with and without statistically adjusting for confounding factors addressing maternal health, socioeconomic status, maternal age, and child sex.

**Results:**

In unadjusted analyses, antibiotic exposure was inversely associated with child socioemotional development. However, after statistically adjusting for important confounds, socioemotional development was not associated with prenatal antibiotic exposure at any dosage or trimester of pregnancy (all *β* ≤ −0.02).

**Conclusion:**

Prenatal antibiotic exposure does not appear to impact early childhood socioemotional development. Maternal health and sociodemographic factors are confounded with antibiotic exposure and socioemotional development, a fact that should be considered in future research examining the effects of prenatal antibiotic exposure on child health. These findings may be reassuring to families who are concerned about the long‐term effects of antibiotics in pregnancy on child health outcomes.

**Key points**

- Many women receive antibiotics during pregnancy. While medically necessary, antibiotics can disrupt the mothers' microbiome, and recent research suggests that it may also alter the infants' microbiome, although very few studies have investigated long‐term outcomes in children exposed to prenatal antibiotics
- Using a large longitudinal dataset, prenatal exposure to antibiotics did not adversely impact child socioemotional outcomes once important confounders like maternal health and sociodemographic factors were taken into account
- Future research should examine whether prenatal antibiotic exposure impacts other domains of development, and include high‐risk populations

## INTRODUCTION

A healthy microbiome is essential for infant health, helping to protect against harmful microbes, regulate metabolism, and improve homeostasis of the immune system (Guarner & Malagelada, 2003). The microbiome is the ecosystem of microorganisms that live in and on humans, often estimated to outnumber human cells 1:10 (Turnbaugh et al., 2007). Recent research has demonstrated that prenatal antibiotics can impact the development of the infant microbiome (Arboleya et al., 2015; Martinez de Tejada, 2014). For example, infants exposed to antibiotics in utero have antibiotics in their first bowel movement (meconium), suggesting that antibiotics in maternal circulation can cross the placental barrier to effect the fetal gastrointestinal tract (Zhao et al., 2019). Moreover, infants of mothers administered pre‐labor antibiotics show gut microbial changes lasting up to 12 months compared to infants whose mothers did not take antibiotics (Azad et al., 2016). Antibiotics also significantly alter the gut‐microbiome of the mother (the primary consumer) which may impact infant health indirectly given that lack of maternal microbiome diversity is associated with fetal inflammation and metabolic dysfunction (Koren et al., 2012; Langdon et al., 2016; Lin et al., 2003). Given that antibiotics in pregnancy alter the infant and maternal microbiome, it is perhaps not surprising that prenatal antibiotic exposure is associated with higher rates of childhood asthma and an 84% higher risk of childhood obesity (Mueller et al., 2015; Stensballe et al., 2013). Antibiotics alter the gut microbiome in powerful ways, and subsequently affect multiple systems of the body.

For example, emerging research suggests that the gut microbiome plays an important role in shaping brain development and, by doing so, socioemotional development (Desbonnet et al., 2014; Mayer et al., 2015). For instance, germ‐free mice, who lack a gut microbiome, commonly exhibit permanent neurological deficits (Luczynski et al., 2016), including neural alterations on brain areas important for social cognition (Arentsen et al., 2015; Selkrig et al., 2014). Accordingly, germ‐free mice display social deficits, some of which normalize after bacterial colonization of the gut, while others persist even after colonization (Moya‐Pérez et al., 2017). Moreover, rat mothers given antibiotics before and during pregnancy have offspring with a 50% decreased interest in social interactions compared to non‐exposed rats (Degroote et al., 2016). Very few studies have investigated how early antibiotic exposure impacts social development in humans. One longitudinal study of 342 children followed from birth to 11 years of age found that exposure to antibiotics in the first 6 months life was associated with significantly lower cognitive and verbal skills at 11 years of age, as well as problems with behavior, attention, anxiety, and emotional regulation (Slykerman et al., 2019).

Together, these animal and human studies suggest that there may be critical periods during which time the microbiome impacts brain regions important for socioemotional development. Theoretically, then, exposure to factors that alter the gut microbiome early in development, like exposure to prenatal antibiotics, may impact offspring socioemotional development. Given that socioemotional competence in early childhood can have a lifelong impact on behavior and health, identifying factors that shape socioemotional development could help improve child emotional, social, and academic outcomes (Briggs‐Gowan & Carter, 2008; Campbell et al., 2006; J. Cohen et al., 2005; Denham, 2006). At the time of writing, we could find no studies evaluating the effects of prenatal antibiotic exposure on childhood socioemotional development in humans, despite the fact that at least 40% of mothers in the United States take prenatal antibiotics (Ledger & Blaser, 2013). Therefore, this study evaluated the associations between antibiotic exposure in utero and children's socioemotional development in early childhood through age 4.5 years using a large, nationally representative New Zealand dataset.

## METHODS

### Participants

This study utilized data from the Growing Up in New Zealand (GUiNZ; www.growingup.co.nz) longitudinal cohort study. GUiNZ enrolled a cohort of 6853 pregnant mothers between 2009 and 2010. The team collected data in the last trimester of pregnancy, and postnatally at 9 months, 2 years, and 4.5 years of child age. Pregnant women were recruited by health professionals or GUiNZ recruiters in medical and community settings (Morton et al., 2014). The enrolled sample is representative of the New Zealand population on key sociodemographic and ethnic characteristics (see Table 1). For example, report this sample is ethnically diverse, with the top five represented ethnicities being 60.9% European, 12.8% Māori, 11.6% Asian, 11.4% Pacific, and 1.6% Middle Eastern, Latin American and African. Full details about the GUiNZ study are reported in Morton et al. (2014, 2017).

**TABLE 1:** Sample characteristics and their association with primary study outcomes

	Descriptive statistics	Any antibiotic exposure	Total antibiotic courses	Socioemotional development
	*M* (*p* value)	β (*p* value)	*β* (*p* value)	*β* (*p* value)
Child male sex %^a^	50.5 (male)	−0.093 (0.061)	−0.012 (0.031)	0.223 (0.028)
Deprivation quantile^b^	3.12 (1.422)	0.084 (0.023)	0.050 (0.011)	−0.079 (0.010)
Maternal health	2.70 (0.971)	−0.192 (0.033)	−0.126 (0.017)	0.116 (0.015)
Maternal education	2.26 (1.140)	−0.143 (0.029)	−0.088 (0.15)	0.062 (0.013)
Maternal age	30.44 (5.821)	−0.024 (0.006)	−0.014 (0.003)	0.017 (0.003)

^a^ Boy = 1, girl = 2.

^b^ Higher score indicates higher deprivation area.

All GUiNZ participants who had socioemotional and antibiotic data available were included in the analyses for the current study. Out of the children who participated in the GUiNZ study, prenatal antibiotic data were not available for 2.2% of the sample and complete socioemotional data were not available for 21%. While study retention at 4.5 years was 90% of the baseline, the missing data can be largely attributed to ability to opt out of answering socioemotional development questions, time limitations during interviews, and participants skipping one of the waves of data collection (S. Morton et al., 2017; Peterson et al., 2019). The current study focuses on the 4800 children who had data on both prenatal antibiotic exposure and socioemotional development in early childhood. We explored how families that were included in our analyses differed from families that did not have available data using linear regression analysis (see Table S1 in the Supporting Information for details). Although the means of the groups were similar, families that were included in our analyses were more likely to live in lower deprivation areas (measured using an area level composite score), had higher maternal age and education, and better maternal health, than those who did not have available data (Salmond et al., 2007). The implications of these missing data are discussed in the limitations section.

This study was approved by the Ministry of Health Northern Y Regional Ethics Committee (NTY/08/06/055), and informed consent was obtained from primary caregivers for use of interview and linked prenatal antibiotics use data.

### Measures

#### Antibiotic exposure

Information regarding exposure to antibiotics was determined by linkage of the GUiNZ participant data with the Pharmaceutical Collection*,* a national dataset that includes all prescription medication dispensed by pharmacists in New Zealand (*Pharmaceutical Collection*, n.d.). A full description of the collection of antibiotics data for the GUiNZ study are reported in Hobbs et al. (2017). Briefly, all antibiotics for systemic use were only available with a prescription in New Zealand at the time of data collection, and each dispensing was recorded in the Pharmaceutical Collection and stored by the Ministry of Health (Horsburgh et al., 2009). Any antibiotics dispensed on a single day were counted as a single dose, while any antibiotics dispensed on separate days were counted as separate doses, regardless of time between dispensing. Any prenatal antibiotic exposure (0 = none; 1 = antibiotic exposure) was examined across all of pregnancy and by trimester of pregnancy. We also examined the number of courses of antibiotics mothers took during pregnancy (hereafter referred to as *antibiotic courses*) and during each trimester.

#### Child socioemotional development

Given that socioemotional development is a multifaceted construct, socioemotional development was measured across the early childhood period using a composite index developed by the GUiNZ team. Full details regarding how the socioemotional development index was developed and validated can be found in Peterson et al. (2019). Briefly, the index utilizes 2626 variables from seven measures taken over three timepoints in early childhood, at 9 months, 2 years, and 4.5 years. At 9 months of age, the composite included scores from the Infant Behavior Questionnaire (Putnam et al., 2014). At 2 years, scores were included from the Strengths and Difficulties Questionnaire (Goodman, 1997), sections of the MacArthur Cognitive Development Inventory – Toddler Short Form (Fenton et al., 2000), and the DesRosier Measure of Self‐Concept (DesRosiers & Busch‐Rossnagel, 1997). At 4.5 years, scores were included from the Children's Behavior Questionnaire‐Very Short Form (Putnam & Rothbart, 2006), parts of the Anticoagulation Knowledge Tool (Denham, 1986), and from GUiNZ team interviewer structured observations. This enabled the assessment of emotional expression and emotional understanding at 9 months, and 2 and 4.5 years; regulation of emotions and behavior at 2 and 4.5 years; and social problem solving and social relationship skills at 4.5 years. Extensive analyses led to the selection of one parsimonious socioemotional development index that showed satisfactory internal consistency and ability to differentiate between poor and high performers on assessment of inhibitory control, executive functioning, pragmatic language, and parental ratings of school readiness at 6 years (Peterson et al., 2019). Questionnaire scores were equally weighted and *z*‐scored. Socioemotional scores ranged from 35 to 147 (*M* = 100.05; SD = 14.98), with higher scores indicating higher socioemotional development. The index allows for the use of SD to categorize levels of socioemotional development, however, in our analysis, we used this index as a continuous score to highlight the spectrum of socioemotional development.

#### Confounders

Confounders were selected because they had been shown to predict either antibiotic use or child socioemotional development in previous research (Palmer et al., 2013; Stokholm et al., 2013), including: child sex, neighborhood deprivation index, maternal age, maternal education level, and general maternal health. All confounder data were collected during face‐to‐face interviews with mothers during pregnancy, aside from child sex, which was collected via face‐to‐face interviews with mother and partner at 9 months postpartum. The deprivation index is based on the New Zealand Index of Deprivation (Salmond et al., 2007), an area‐based measure of socioeconomic deprivation in New Zealand neighborhoods using Census data (Atkinson et al., 2014). In this index, composite scores are based on nine socioeconomic variables, and range from 1 to 10. Quantile 1 represents areas with the 10% least deprivation, and quantile 10 represents areas with the 10% most deprivation (Atkinson et al., 2014). In order to measure maternal age at antenatal interview, mothers were asked to provide their date of birth. To assess maternal education, mothers were asked “what is your highest completed secondary school qualification?”, scores ranged from 0 to 4, where 0 = no secondary school qualification, 1 = secondary school, 2 = diploma/trade certificate, 3 = Bachelor's degree, and 4 = higher degree. Maternal health was self‐ranked on a scale from 1 to 5, with 5 being excellent health and 1 being poor health.

### Statistical strategy

We employed a three‐step statistical strategy. First, unadjusted linear regression models were used to test whether antibiotic exposure in pregnancy predicted childhood socioemotional development. Second, we constructed multivariable linear regression models using antibiotic exposure and socioemotional development as outcomes to identify potential sociodemographic confounders. Any confounder that significantly predicted either antibiotic exposure or socioemotional development in multivariable models was examined as a potential confounder (*p* < 0.10). Finally, we used multivariable models to examine whether any observed association between antibiotic exposure in pregnancy and socioemotional development remained statistically significant after adjusting for potential confounders. Linear regression was used in cases of continuous outcome variables (i.e., socioemotional development, total antibiotic courses) and binominal logistic regression was used in the case of dichotomous outcomes (i.e., any antibiotic exposure). All analyses were performed in SPSS version 19.0. Given the large sample size, antibiotic effects were considered statistically significant with *p* < 0.01.

## RESULTS

### Descriptive statistics

Table 1 presents the demographic characteristics. Of the 4800, 1850 (38.5%) women received antibiotics in pregnancy, ranging from one antibiotic course to 11 courses, with the women exposed to antibiotics averaging 1.70 courses (SD = 1.13). 16.4% of women took antibiotics in the first trimester, 14.3% in the second trimester, and 18.6% in the third trimester.

### Unadjusted associations

Table 2 presents results for the unadjusted, univariate regression models**,** showing that any antibiotic exposure, both at any time in pregnancy and in the first and third trimesters separately, were significantly associated with reduced child socioemotional development scores. Moreover, antibiotic courses at any time in pregnancy and in each trimester were also significantly associated with child socioemotional development in unadjusted models (see Table 2).

**TABLE 2:** Linear regression models of the association between antibiotics in pregnancy and child socioemotional development

	Throughout pregnancy		First trimester		Second trimester		Third trimester	
	*β*	*p* value	*Β*	*p* value	*β*	*p* value	*β*	*p* value
Any antibiotic use								
Unadjusted	−0.072	**0.000**	−0.047	**0.001**	−0.037	0.011	−0.053	**0.000**
Adjusted	−0.020	0.149	−0.007	0.622	−0.001	0.933	−0.015	0.278
Antibiotic courses								
Unadjusted	−0.076	0.000	−0.051	**0.000**	−0.035	0.014	−0.063	**0**.**000**
Adjusted	−0.017	0.241	−0.011	0.420	0.003	0.850	−0.022	0.223

*Note*: Unadjusted models include just the antibiotic variable predicting socioemotional development; adjusted models include the antibiotic variable along with child sex, maternal age, maternal health, maternal education, and deprivation quantile (a marker of socioeconomic status). *p* < 0.01 is marked in bold.

### Confounder analyses

As shown in Table 1, deprivation quantile, maternal health, maternal education, and maternal age were all significantly associated with both antibiotic exposure and socioemotional development scores. Child sex was associated with socioemotional development, in that boys tended to score slightly lower than girls. Therefore, deprivation quantile, maternal health, maternal education, maternal age, and child sex were included in multivariate regression models along with antibiotic exposure to predict child socioemotional development.

### Adjusted associations

As detailed in Table 2 (Model 2), after statistically adjustment for sociodemographic confounders, none of the prenatal antibiotic exposure variables were associated with child socioemotional development.

## DISCUSSION

The aim of this study was to test for an association between prenatal antibiotic exposure and early child socioemotional development in humans. Before controlling for important confounders, there was an inverse relationship between prenatal antibiotic exposure and socioemotional developmental overall in pregnancy as well as in the first and third trimesters. However, after adjusting for these confounders, including deprivation quantile, maternal health, maternal education, maternal age, and child sex, there was no longer a statistically significant association between prenatal antibiotic exposure and child socioemotional development. Thus, we discern that socioemotional development was not adversely impacted by typical prenatal antibiotic exposure in this nationally representative, non‐clinical sample. Our null results are consistent with other large population based studies that show no clinically significant association between antibiotic exposure in early childhood and socioemotional disorders like autism spectrum disorder (Hamad et al., 2018).

These null results may be reassuring to the growing number of women who are reluctant to take antibiotics in pregnancy, despite the fact that prenatal bacterial infection is dangerous for the fetus and is the most common cause of stillbirth in developing countries (Goldenberg & Thompson, 2003). Nationally representative studies like this one that rigorously examine the long‐term developmental implications of early antibiotic may help to assuage fears and reduce antibiotic refusal, which can be deadly for mothers and babies (Bauchner et al., 1999; M. L. Cohen, 1992). Future research is still warranted to determine whether prenatal antibiotics have impacts in other child domains (e.g. cognitive abilities and infant microbiome diversity), in clinical populations, or when used in extreme doses.

Our results suggest that caution should be used before extrapolating the results from rodent studies of prenatal antibiotic exposure directly to humans, especially given that the dosages are often quite different. For example, in a rodent study that examined the impact of prenatal antibiotic exposure on offspring behavior, 7‐day courses of antibiotics were administered to pregnant mice (Tochitani et al., 2016). While this course length aligns with the typical adult human antibiotics course of 5–7 days, mice and human gestation periods are vastly different. A 7‐day course of antibiotics in humans is only 2.5% of a typical 40‐week pregnancy, while the same 7‐day course makes up 37.8% of the mouse gestational period, equivalent to approximately 15 weeks of a human pregnancy. The average number of antibiotic courses in the current cohort was comparatively low (*M* = 1.70). To make a more direct comparison to rodent studies, future observational research could be conducted with higher risk populations, such as pregnant women with chronic bacterial infections that require frequent antibiotics throughout pregnancy. Another reason why our results may differ from those of animal studies is that it is difficult to do experimental paradigms on antibiotic use in humans and, as our study shows, antibiotic use in humans is confounded with sociodemographic outcomes and maternal health.

For example, we found that sociodemographic variables were associated with early antibiotic exposure, similar to previous studies (Thrane et al., 2003). Mothers who live in more deprived neighborhoods are more likely to have children who are prescribed antibiotics (Hobbs et al., 2017). This association may be due to the fact that poverty is associated with poorer maternal health and nutrition, both which increase the risk of infection (Thrane et al., 2003). Consistent with previous research, our findings showed an inverse relationship between maternal age and antibiotic exposure (Stokholm et al., 2013). One possible explanation is that increased maternal age is associated with psychological, economic, and educational advantages, leading to better physical health, and a lesser need for antibiotics (Trillingsgaard & Sommer, 2018). It is notable that we still found a modest correlation between neighborhood deprivation and antibiotic exposure in this New Zealand sample, despite the fact all participants do have access to affordable healthcare during their pregnancy regardless of sociodemographic factors. This suggests that other structural inequalities beyond health care access may play a role, such as maternal stress during pregnancy, nutrition, and education. Still, we speculate that the relationship between the poverty and antibiotic exposure would be even stronger in nations without access to universal healthcare like the United States (Kim et al., 2018).

This study has several strengths including the use of a large and diverse cohort with high levels of retention, and a thorough socioemotional development index. Another major strength is access to pharmaceutical records of prenatal antibiotic use, which allowed for accurate quantification of antibiotics without reliance on participant recall. Actual attrition across the 5‐year follow‐up period, where 90% of enrolled participants completed the assessment at age 4.5 years, compares favorably to other longitudinal studies (Teague et al., 2018). However, this study also has several limitations. First, as is the case for most multiyear longitudinal studies, about a fifth of participants were either lost to follow‐up or did not have available data to calculate the socioemotional composite score due to time constraints of interviews and the extensive nature of the questionnaires. Second, although the GUiNZ team oversampled populations that were more likely to drop from the study, it is important to note that the participants that remained in our cohort were healthier and of higher socioeconomic status than those that left the study, potentially making these findings less representative of the general population. Additionally, the information on the Pharmaceutical Collection does not include antibiotics dispensed directly by a medical practitioner in an emergency situation, nor does it include if the course was completed (Horsburgh et al., 2009). Finally, we did not examine whether exposure to specific classes of antibiotics (e.g., amino penicillin vs. macrolides vs. penicillin) was associated with child socioemotional outcomes differently and future hypothesis‐driven research in this area may be warranted.

In sum, we conclude that prenatal exposure to antibiotics does not appear to be adversely associated with child socioemotional development. Long‐term child psychological outcomes have often been neglected in the study of the effects of prenatal medical interventions, inadvertently leading to parental speculation and anxiety. These findings may be a reassurance to women who are concerned about the effects of antibiotics in pregnancy. Additional large‐scale studies that test the long‐term physiological impact of antibiotics and other prenatal interventions will be important moving forward to head‐off unfounded speculation and ensure that antibiotic hesitancy does not impede infant health.

## CONFLICT OF INTEREST

The authors have declared that they have no competing or potential conflicts of interest.

## ETHICAL CONSIDERATIONS

This study was approved by the Ministry of Health Northern Y Regional Ethics Committee (NTY/08/06/055), and informed consent was obtained from primary caregivers for use of interview and linked prenatal antibiotics use data.

## AUTHOR CONTRIBUTIONS

Adi Fish‐Williamson conceptualized the research question, primarily wrote the manuscript, and performed statistical analyses. Dr. Jan Wallander contributed to data interpretation and assisted in manuscript preparation. Dr. Mark Hobbs assessed with data‐processing and consulted on research design. Dr. Susan M. B. Morton oversaw data‐collection. Dr. Jennifer Hahn‐Holbrook closely oversaw all statistical analyses and manuscript writing. All authors contributed to revisions of the manuscript and have approved the final manuscript.

## Supporting information

TABLE S1

## Data Availability

The data and material used in this study are available on request from the Growing Up in New Zealand study (please see https://www.growingup.co.nz/using‐data, contact dataaccess@growingup.co.nz), and Pharmaceutical Collection of the New Zealand Ministry of Health (please see https://www.health.govt.nz/nz‐health‐statistics/national‐collections‐and‐surveys/collections/pharmaceutical‐collection, contact data‐enquiries@health.govt.nz). The data are not publicly available due to containing information that could compromise research participant privacy and consent.
