# Supplementary material for: Prenatal antibiotic exposure in pregnancy and early childhood socioemotional development
Source: JCPP Adv. 2022 Mar 29;2(2):e12066. doi: 10.1002/jcv2.12066 (PMC10242931; doi:10.1002/jcv2.12066)
Supplement: Supplementary file 1 — TABLE S1 [file JCV2-2-e12066-s001.docx]

| **Cohort** | | | | | **Non-Cohort** | | | |
| --- | --- | --- | --- | --- | --- | --- | --- | --- |
|  | *N* | *Minimum* | *Maximum* | *Mean (SD)/%* | *N* | *Minimum* | *Maximum* | *Mean (SD)%* |
| Gender (% Male)* | 4800 | - | - | 50.5% | 1136 | - | - | 54% |
| Neighborhood Index* | 4800 | 1 | 5 | 3.120 (1.422) | 1271 | 1 | 5 | 3.280 (1.466) |
| Maternal Age | 4800 | 15 | 47 | 30.440 (5.821) | 1453 | 15 | 46 | 30.230 (5.796) |
| Maternal Education* | 4800 | 0 | 4 | 2.260 (1.140) | 1453 | 0 | 4 | 2.100 (1.201) |
| Maternal Health* | 4800 | 0 | 9 | 2.700 (0.971) | 1453 | 0 | 9 | 2.610 (0.976) |

**Table S1. Differences between missing and non-missing participants**

*Covariates indexed with a * denote significant differences between the Cohort and Non-Cohort groups with p <.05*
